# Iterative Reweighted ℓ_1_ Synthesis of Sparse Antenna Arrays with Continuous Element Positions

**DOI:** 10.3390/mi17080922

**Published:** 2026-07-30

**Authors:** Xin-Yu Duan, Wei-Zong Li, Yi-Xuan Zhang, Ye Hui

**Affiliations:** 1 National Key Laboratory of Radar Detection and Sensing, Xidian University, Xi’an 710071, China; duanxy@stu.xidian.edu.cn (X.-Y.D.); wzli_1@stu.xidian.edu.cn (W.-Z.L.); 2 Shaanxi Key Laboratory of Antenna and Control Technology, 39th Research Institute of CETC, Xi’an 710065, China; xyyeah1994@126.com

**Keywords:** sparse array synthesis, reweighted ℓ_1_ norm minimization, convex optimization, position perturbation, reconfigurable arrays, planar arrays, directivity constraint, microwave front ends

## Abstract

Sparse antenna arrays are attractive for compact microwave and millimeter-wave front ends because they can achieve prescribed radiation performance with fewer radiating elements, thereby reducing the number of feeding channels, hardware cost, weight, and power consumption. However, the joint optimization of element positions and complex excitations remains challenging, since the element positions enter the array factor nonlinearly and the element-count objective is inherently combinatorial. This paper presents an iterative reweighted ℓ_1_ synthesis framework for sparse antenna arrays with continuous element positions. At each iteration, position perturbations are introduced and the array factor is linearized using a first-order Taylor expansion within a trust region. The resulting non-convex sparse synthesis problem is then approximated by convex programing through an iteratively reweighted ℓ_1_ relaxation, allowing the excitation amplitudes, phases, and element positions to be updated simultaneously. Additional aperture, minimum-spacing, and minimum-directivity requirements are formulated as convex constraints and incorporated when required, enabling joint control of sparsity, sidelobe level, physical layout, and radiation performance. The proposed method is validated through four representative examples, including a shaped-beam linear array, a tri-pattern reconfigurable linear array, a planar pencil-beam array, and a directivity-constrained planar array. Compared with fixed-grid reweighted ℓ_1_ methods under the same specifications, the proposed approach produces sparser layouts while avoiding the grid-resolution limitation. In the directivity-constrained benchmark, it also achieves competitive element reduction while enforcing a prescribed minimum element spacing. These results indicate that the proposed framework provides a flexible and practical synthesis tool for compact and integrated sparse antenna-array design.

## 1. Introduction

Antenna arrays with many radiating elements are key components of modern radar, satellite communication, remote sensing, and millimeter-wave systems, where they provide the high gain, narrow beams, and rapid electronic scanning that these applications demand. In a conventional periodic array, the inter-element spacing is fixed to about half a wavelength to avoid grating lobes, so the number of elements, and the associated transmit/receive modules, phase shifters, and feeding channels, grows rapidly with the aperture. Sparse (aperiodic) arrays break this periodicity and exploit the element positions as additional degrees of freedom, realizing a prescribed pattern with significantly fewer elements [1,2] and thereby cutting the cost, weight, power consumption, and heat dissipation of the front end. This feature is particularly attractive for compact and integrated microwave or millimeter-wave array front ends, where the number of feeding channels, available aperture, and element spacing are often tightly constrained.

However, determining the minimum number of elements, together with their positions and complex excitations, that satisfies a prescribed pattern mask is a doubly non-convex problem: the positions enter the array factor nonlinearly, while the element-count objective is the ℓ_0_ norm. Early studies addressed this difficulty mainly with stochastic global optimizers, such as genetic algorithms, simulated annealing, differential evolution, and related evolutionary schemes [3,4,5], and with deterministic methods such as iterative Fourier techniques [6,7] and the matrix pencil method [8]. Although effective for moderate problem sizes, the computation time of these methods grows steeply with the number of unknown variables, which makes them difficult to apply to large sparse arrays or largely confines them to specific array categories, resulting in limited generality.

The advent of convex optimization [9,10] reshaped this picture by recasting array synthesis as a problem that can be solved both efficiently and reproducibly. Building on the reweighted ℓ_1_ minimization principle [11], a family of convex formulations, including sequential convex programming [12,13] and antenna-selection or sparseness-constrained models [14,15], was developed to minimize the element count under arbitrary pattern masks. The convex paradigm has since been refined toward higher efficiency and broader generality, for instance through first-order iterative convex approximation [16] and sequential convex optimization under bilateral (two-sided) mask constraints [17].

Existing sparse array synthesis methods nevertheless rely predominantly on fixed spatial grids whose resolution is inherently limited: a coarse grid misses the optimal off-grid positions, whereas a dense grid leads to ill-conditioning and sub-wavelength clustering and provides no convex control over element spacing [18]. Although off-grid perturbation [18,19] and steerable convex designs [20] alleviate this grid dependence, they still cannot impose a guaranteed minimum spacing. These bottlenecks extend to more demanding scenarios: in multiple-pattern synthesis, existing methods [21,22,23,24] remain hindered by similar grid restrictions, whereas directivity-aware techniques that enforce a gain floor [20,25,26,27,28] still lack continuous position control. Consequently, a unified convex framework is still needed which can jointly and continuously optimize the positions and excitations of single-pattern, multiple-pattern, and planar arrays while systematically incorporating minimum-spacing and directivity constraints within a tractable convex-optimization procedure.

In this paper we propose such a unified joint convex-optimization framework. At each iteration a position-perturbation variable is introduced and the array factor is linearized by a first-order Taylor expansion under a trust-region constraint; the bilinear excitation–perturbation coupling is decoupled using the previous excitation estimate, and the ℓ_0_ objective is relaxed to an iteratively reweighted ℓ_1_ norm, so that each iteration is a second-order-cone program that jointly updates the element number, positions, and complex excitations; aperture and minimum-spacing bounds are appended as convex constraints only when the application requires them. To validate the versatility of this framework, our contributions are structured across four progressively complex scenarios: (1) single-pattern linear arrays, establishing a baseline convex model that guarantees strict minimum element spacing; (2) multiple-pattern linear arrays, introducing a reweighted ℓ_2,1_ group-sparsity objective to enforce a shared physical layout for pattern-reconfigurable systems; (3) planar sparse arrays, extending the framework to 2D coordinates by convexifying the highly non-convex pairwise minimum-distance constraints; and (4) directivity-constrained arrays, incorporating a minimum-directivity requirement as a convex quadratic constraint to prevent gain degradation. The remainder of the paper is organized as follows: Section 2 formulates the synthesis problem, Section 3 develops the proposed method and its directivity-constrained variant, Section 4 reports the numerical results, and Section 5 concludes.

## 2. Problem Formulation of Linear and Planar Sparse Arrays

### 2.1. Array Models

Consider an array of N elements located at continuous positions $r_{n}$, where n=1,…,N, with complex excitations $w_{n}=A_{n}e^{j\varphi_{n}}$ collected in the vector $w={\left[w_{1},\ldots ,w_{N}\right]}^{T}$. The far-field array factor at the observation angle θ is

(1) $$AF\left(\theta \right)\;=\;\sum_{n=1}^{N}w_{n}\;e^{jk\cdot r_{n}}\;=\;a{\left(\theta \right)}^{H}\;w,$$

where k denotes the wavenumber vector, $a\left(\theta \right)={\left[e^{jk\cdot r_{1}},\ldots ,e^{jk\cdot r_{N}}\right]}^{H}$ is the steering (direction) vector, ${\left(\cdot \right)}^{T}$ denotes transposition, and ${\left(\cdot \right)}^{H}$ denotes the conjugate transpose, respectively.

The compact form ${a\left(\theta \right)}^{H}w$ separates the two ingredients of the radiated pattern: the excitations w, which serve as the design variables, and the steering vector $a\left(\theta \right)$, which encodes how the element positions $r_{n}$ shape the phase front in each observation direction.

The steering vector takes a specific form once the array geometry is fixed. For a linear array along the x-axis, the position of each element reduces to the scalar $r_{n}$ and the steering phase is

(2) $$k\cdot r_{n}\;=\;\frac{2\pi }{\lambda }\;r_{n}sin\theta ,$$

with λ being the wavelength. For a planar array in the xy plane, the position of the n-th element is the vector $r_{n}={\left(x_{n},y_{n}\right)}^{T}$, the observation direction is described by the elevation–azimuth pair $\left(\theta ,\phi \right)$, and the steering phase becomes

(3) $$k\cdot r_{n}\;=\;\frac{2\pi }{\lambda }\left(x_{n}sin\theta cos\phi +y_{n}sin\theta sin\phi \right).$$

The two geometries therefore share the same compact array factor expression (1) and differ only in the dimension of the position variable—one coordinate per element for the linear array, and two for the planar array. This shared structure is precisely what allows the linear, multiple-pattern, and planar versions of the proposed method to be developed within a single framework in Section 3.

### 2.2. Highly Sparse Synthesis Problem

In a practical array, the cost, weight, and feed-network complexity grow with the number of radiating elements, which motivates synthesizing a prescribed pattern with as few elements as possible. Let $F_{d}\left(\theta \right)$ denote the desired pattern in the mainlobe region, and $\Theta_{ML}$ and $\Theta_{SL}$ the sets of sampling angles in the mainlobe and sidelobe regions, respectively. These samples are taken over the one-dimensional visible region for linear arrays and over the two-dimensional region $\left(\theta ,\phi \right)$ for planar arrays, so that the formulation below applies to both geometries without modification. Two scalars specify the admissible pattern mask: ε bounds the ripple by which the synthesized mainlobe may deviate from the desired pattern, and ρ bounds the sidelobe level.

A radiating element is inactive precisely when its excitation is zero, so the number of active elements equals the number of non-zero entries of the excitation vector. The highly sparse synthesis problem is therefore to minimize this count subject to the pattern mask:

(4) $$\underset{w,\;r}{min}\;\;∥w\;{∥}_{0}\;s.t.\;\;\;\;\left|AF\left(\theta_{i}\right)-F_{d}\left(\theta_{i}\right)\right|\leq \varepsilon ,\;\;\;\;\theta_{i}\in \Theta_{ML},\;\left|AF\left(\theta_{j}\right)\right|\leq \rho ,\;\theta_{j}\in \Theta_{SL}.$$

where $∥\;{∥}_{0}$ counts the non-zero excitations, and the optimization is carried out jointly over the excitations w and the element positions r.

Problem (4) is doubly non-convex. First, the $l_{0}$ “norm” in the objective is non-convex and combinatorial in nature. Second, the positions r appear in the complex exponentials $a\left(\theta \right)$, so the mask constraints depend non-convexly on the positions. The proposed method addresses the two difficulties separately: the position dependence is handled by a trust-region linearization, and the $l_{0}$ objective is relaxed through an iteratively reweighted $l_{1}$ minimization, as detailed in Section 3.

## 3. Proposed Iterative Reweighted Synthesis Method

**Figure 1:**
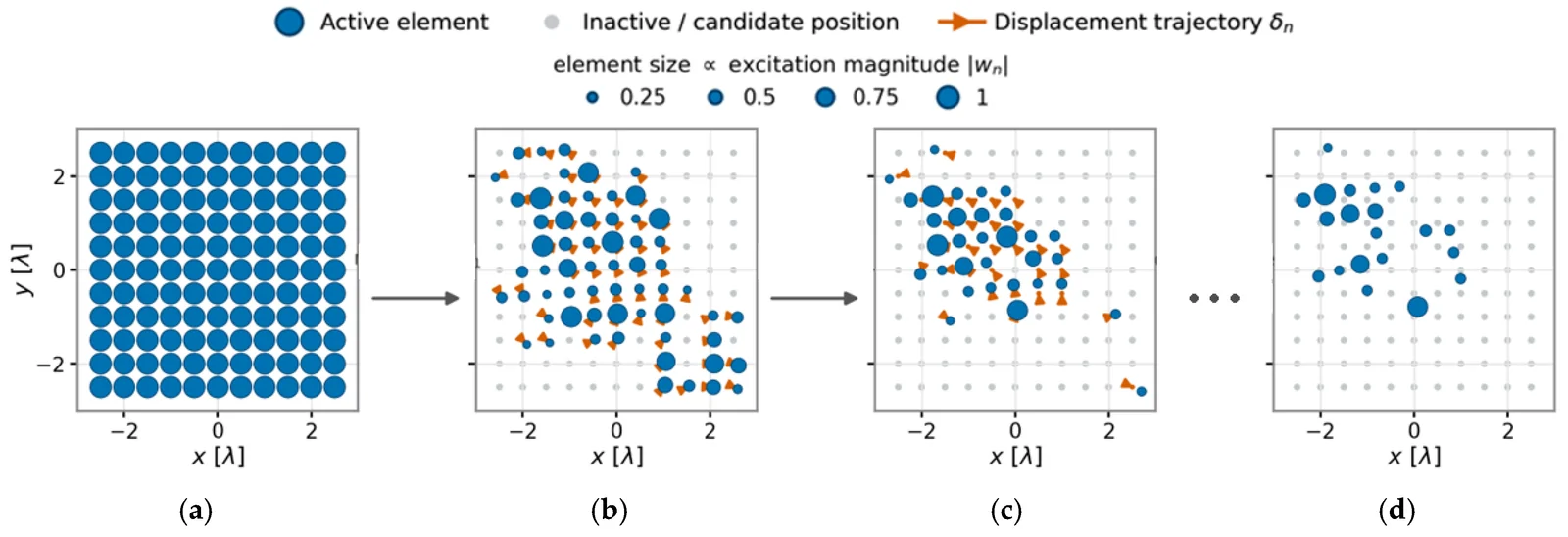
Illustration of the joint evolution of the array layout and excitation during the iterative sparse array synthesis. The marker area is proportional to the excitation magnitude |w_n_|, blue and gray markers denote active elements and inactive (candidate) positions respectively, and the orange arrows indicate the element-position displacement trajectory δ_n_. At each iteration the element excitations and positions are perturbed jointly. (**a**) Initial fully populated array on the candidate grid with uniform excitation; (**b**) array after the first iteration, where the excitations and positions are jointly updated and parts of the elements are switched off; (**c**) array at the second iteration with the number of active elements further reduced; and (**d**) the converged array at the final iteration, yielding the sparsest layout that satisfies the pattern requirement.

In this section, the superscript (G) denotes the iteration index. To better illustrate the proposed algorithm, a planar array is taken as an example for demonstration, as shown in Figure 1. As shown in Figure 1a, the synthesis starts from a densely sampled candidate grid on which all elements are active and uniformly excited. Within a single iteration, the proposed method perturbs the element excitations and positions jointly: the excitation magnitudes |w_n_| are reweighted to penalize weakly excited elements and gradually drive them toward zero, while the surviving elements are concurrently displaced along the trajectory δ_n_ to better approximate the desired pattern. Consequently, after the first iteration [Figure 1b], the excitations and positions are jointly updated and a portion of the elements is switched off. As the iterations proceed [Figure 1c], the number of active elements keeps decreasing while the remaining excitations and positions are progressively refined together. This process is repeated until convergence [Figure 1d], yielding a highly sparse array layout that satisfies the prescribed pattern constraints. Owing to this joint perturbation strategy, the coupling between excitation and geometry is fully exploited, so that the element number can be effectively reduced and the array layout is optimized in a unified manner.

Building upon this joint perturbation framework, the proposed method is applied to four progressively complex scenarios, namely single-pattern linear arrays, multiple-pattern linear arrays, planar sparse arrays, and directivity-constrained arrays. These cases extend the framework from a basic linear model to multiple-pattern, two-dimensional, and directivity-constrained synthesis with increasing modeling complexity. Together they verify that the proposed method can flexibly accommodate diverse synthesis requirements while consistently reducing the number of array elements.

### 3.1. Sparse Linear Arrays

At iteration G, a perturbation matrix $\delta^{\left(G\right)}$ of the element positions is introduced, so that the new positions are $r_{n}^{\left(G\right)}+\delta_{n}^{\left(G\right)}$. Applying a first-order Taylor expansion of the steering phase at the current position matrix $r^{\left(G\right)}$ yields

(5) $$e^{\;jk\cdot \left(r_{n}^{\left(G\right)}+\delta_{n}^{\left(G\right)}\right)}\;\approx \;e^{\;jk\cdot r_{n}^{\left(G\right)}}\left(1+j\;k\cdot \delta_{n}^{\left(G\right)}\right),$$

which is accurate as long as the perturbation stays small. To keep the linearization within this small-perturbation regime, we impose the trust-region (limited-perturbation) constraint

(6) $${∥\delta_{n}^{\left(G\right)}∥}_{\infty }\;\leq \;\beta ,\;\;n=1,\ldots ,N,$$

where β is the position-perturbation upper bound, $\delta_{n}^{\left(G\right)}$ is the perturbation vector of the n-th position in the matrix $\delta^{\left(G\right)}$, and $r_{n}^{\left(G\right)}$ is the n-th position vector of the matrix $r^{\left(G\right)}$; for a linear array they reduce to the scalars $\delta_{n}^{\left(G\right)}$ and $r_{n}^{\left(G\right)}$.

Substituting expansion (5) into array factor (1) produces a term that is bilinear in the unknowns $w^{\left(G\right)}$ and $\delta^{\left(G\right)}$, and this bilinear coupling makes the optimization problem non-convex. To overcome this non-convexity, the excitation in the bilinear term is fixed to its previous-iteration estimate $w^{\left(G-1\right)}$; this approximation is reasonable because the excitations vary slowly between consecutive iterations, and it yields the linearized array factor of the linear array as

(7) $$AF\left(\theta \right)\;\approx \;a^{\left(G\right)}{\left(\theta \right)}^{H}\;w^{\left(G\right)}\;+\;b^{\left(G\right)}{\left(\theta \right)}^{H}\left(w^{\left(G-1\right)}⊙\delta^{\left(G\right)}\right),$$

where ⊙ denotes the element-wise (Hadamard) product and $b\left(\theta \right)={\left[\;jk\;e^{jk\cdot r_{1}},\ldots ,jk\;e^{jk\cdot r_{N}}\right]}^{H}$ is the direction vector with the jk factor introduced by the expansion. The array factor (7) is now jointly affine in $w^{\left(G\right)}$ and $\delta^{\left(G\right)}$, so the pattern mask constraints of (4) become convex (second-order-cone) constraints.

The $l_{0}$ objective of (4), which counts the discrete number of non-zero excitations, is replaced by the weighted sum of the excitation moduli, i.e., a weighted $l_{1}$ norm, whose weights are updated iteratively [11]. The reweighting coefficient vector at iteration G is

(8) $$\alpha^{\left(G\right)}\;=\;1\;/\;\left(\left|w^{\left(G-1\right)}\right|+\eta \;1\right),$$

where the division is performed element-wise, 1 is the all-ones vector which is of the same dimension as w, and η is a small stabilization factor that avoids division by zero. Excitations that are small at the previous iteration receive a large weight and are pushed toward zero, whereas the dominant excitations are penalized only weakly; after a few iterations the excitation vector becomes highly sparse, which emulates the behavior of the $l_{0}$ objective while preserving convexity. Collecting the weighted objective and the linearized pattern constraints together with the trust-region bound, the convex-optimization model solved at iteration G for the linear array is

(9) $$\underset{\delta^{\left(G\right)}\in C^{N\times 1},\;w^{\left(G\right)}\in C^{N\times 1}}{min}\;\;{∥\alpha^{\left(G\right)}⊙w^{\left(G\right)}∥}_{1}\;s.t.\;\;\;\left|a^{\left(G\right)}{\left(\theta_{i}\right)}^{H}w^{\left(G\right)}+b^{\left(G\right)}{\left(\theta_{i}\right)}^{H}\left(w^{\left(G-1\right)}⊙\delta^{\left(G\right)}\right)-F_{d}\left(\theta_{i}\right)\right|\leq \varepsilon ,\;\;\;\theta_{i}\in \Theta_{ML},\;\left|a^{\left(G\right)}{\left(\theta_{j}\right)}^{H}w^{\left(G\right)}+b^{\left(G\right)}{\left(\theta_{j}\right)}^{H}\left(w^{\left(G-1\right)}⊙\delta^{\left(G\right)}\right)\right|\leq \rho ,\;\;\;\theta_{j}\in \Theta_{SL},\;\left|\delta_{n}^{\left(G\right)}\right|\leq \beta ,\;\;\;\;\;\;\;\;n=1,\ldots ,N,$$

where $\Theta_{ML}$ and $\Theta_{SL}$ are the sets of mainlobe and sidelobe sampling angles, $\theta_{i}$ and $\theta_{j}$ are the angles in $\Theta_{ML}$ and $\Theta_{SL}$, $F_{d}$ is the desired mainlobe pattern, N is the total number of candidate elements, n is the element index, ρ is the sidelobe upper bound, ε is the mainlobe ripple bound, and β is the perturbation upper bound. Problem (9) is a second-order-cone program that can be solved efficiently by off-the-shelf solvers such as CVX [29].

It should be noted that the basic model (9) contains only the pattern and trust-region constraints; any further geometric requirement is appended on a per-application basis. When the array must fit within an installation platform of a given size, for example, the perturbed positions are confined to the available aperture of diameter D by the aperture-range constraint

(10) $$\left|r_{n}^{\left(G\right)}+\delta_{n}^{\left(G\right)}\right|\;\leq \;D/2,\;\;n=1,\ldots ,N,$$

and when the physical size of the radiators or mutual-coupling considerations impose a minimum separation d between adjacent elements—whose order along the axis is preserved by the trust-region bound—the minimum-spacing constraint

(11) $$\left(r_{n+1}^{\left(G\right)}+\delta_{n+1}^{\left(G\right)}\right)-\left(r_{n}^{\left(G\right)}+\delta_{n}^{\left(G\right)}\right)\;\geq \;d,\;\;\;\;\;\;\;\;n=1,\ldots ,N-1,$$

is added. Both (10) and (11) are linear in the optimization variables, so appending them to (9) preserves the convexity of the model; if the application imposes no such restrictions, they can simply be omitted.

After solving (9), the element positions are updated according to $r^{\left(G+1\right)}=r^{\left(G\right)}+\delta^{\left(G\right)}$, the reweighting coefficients are updated by (8), and the procedure is repeated. Convergence occurs when the total change in element position between three consecutive iterations, denoted as $\gamma =∥r^{\left(G+1\right)}-r^{\left(G\right)}{∥}_{1}$, drops below the specified accuracy. Elements whose excitation magnitude $\left|w_{n}\right|$ is below the threshold ν are then identified and removed, and the remaining positions, complex excitations, and final radiation pattern are returned. The complete procedure is summarized in Algorithm 1.
**Algorithm 1:** Iterative reweighted *ℓ*_1_ synthesis with joint position–excitation optimization.1: Initialize iteration index *G* = 0, element number *N*, positions ***r***^(*G*)^, excitations ***w***^(*G*)^, reweighting coefficients ***α***^(*G*)^, removal threshold *ν*, precision *η*, and *γ* = ∞ 2: **while**
*G* < *Itr_max_* **do** 3: Solve the convex program via CVX toolbox, and obtain optimal ***w***^(*G*)^ and ***δ***^(*G*)^ 4: Set ***r***^(*G*+1)^ = ***r***^(*G*)^ + ***δ***^(*G*)^ 5: Set *γ* = ‖***r***^(*G*+1)^ − ***r***^(*G*)^‖_1_ 6: Set *G* ← *G* + 1 7: **end while**
8: Return ***r***^(G^*^)^, ***w***^(G^*^)^ after removing elements with |*w_n_*^(*G*)^| < *ν*

Algorithm 1 shows the proposed iterative reweighted l-1 synthesis algorithm with the joint position–excitation optimization process.

### 3.2. Multiple-Pattern Linear Arrays

The single-pattern formulation of Section 3.1 extends directly to systems that must reconfigure their radiation pattern from one shared aperture. Such systems—for instance, a radar that alternates between a pencil beam for tracking and a shaped beam for surveillance—produce several different patterns by switching only the excitations, while the physical layout remains common to all operating modes [21,22,24]. Let Q desired patterns $F_{d}^{\left(q\right)}\left(\theta \right)$, where q=1,…,Q, be specified, each with its own mainlobe set $\Theta_{ML}^{\left(q\right)}$, sidelobe set $\Theta_{SL}^{\left(q\right)}$, ripple bound $\varepsilon_{q}$, and sidelobe bound $\rho_{q}$. The excitation vectors of all the patterns are collected in the matrix $W=\left[w^{\left(1\right)},\ldots ,w^{\left(Q\right)}\right]\in C^{N\times Q}$, whose n-th row $w_{row,n}$ contains the Q excitations of the n-th element.

An element can be removed from the shared layout only if its excitations are zero in all of the patterns simultaneously, i.e., only if the whole row $w_{row,n}$ vanishes. Independent $l_{1}$ penalties on the columns of W would indicate zero different elements in different patterns and would not yield a common sparse layout. The element count is instead measured by the number of non-zero rows of W, which is relaxed into the convex $l_{2,1}$ mixed norm $\sum_{n=1}^{N}∥w_{row,n}{∥}_{2}$, a standard group-sparsity regularizer [23]. Applying the same iterative reweighting principle as in Section 3.1 to the row norms, the objective at iteration G becomes

(12) $$\underset{W,\;\delta }{min}\;\;\sum_{n=1}^{N}\frac{1}{{∥w_{row,n}^{\left(G-1\right)}∥}_{2}+\eta }\;{∥w_{row,n}∥}_{2},$$

where η is the stabilization factor and $w_{row,n}^{\left(G-1\right)}$ is the row estimate of the previous iteration. Rows whose joint energy across all patterns was small are strongly penalized and driven to zero as a group, so the corresponding elements are removed from every operating mode at once.

Because all the patterns share the same physical layout, a single perturbation variable $\delta^{\left(G\right)}$ is used, and the Taylor-expanded array factor (7) is imposed separately for each pattern with its own excitation column. The convex model solved at iteration G for the multiple-pattern linear array is

(13) $$\underset{\delta^{\left(G\right)},\;W^{\left(G\right)}}{min}\;\;\;\sum_{n=1}^{N}\alpha_{n}^{\left(G\right)}{∥w_{row,n}^{\left(G\right)}∥}_{2},\;\;\;\;\;\;\;\;\;\alpha_{n}^{\left(G\right)}=\frac{1}{{∥w_{row,n}^{\left(G-1\right)}∥}_{2}+\eta }\;s.t.\;\;\;\left|a^{\left(G\right)}{\left(\theta_{i}\right)}^{H}w^{\left(q,G\right)}+b^{\left(G\right)}{\left(\theta_{i}\right)}^{H}\left(w^{\left(q,G-1\right)}⊙\delta^{\left(G\right)}\right)-F_{d}^{\left(q\right)}\left(\theta_{i}\right)\right|\leq \varepsilon_{q},\;\;\;\theta_{i}\in \Theta_{ML}^{\left(q\right)},\;\left|a^{\left(G\right)}{\left(\theta_{j}\right)}^{H}w^{\left(q,G\right)}+b^{\left(G\right)}{\left(\theta_{j}\right)}^{H}\left(w^{\left(q,G-1\right)}⊙\delta^{\left(G\right)}\right)\right|\leq \rho_{q},\;\;\;\theta_{j}\in \Theta_{SL}^{\left(q\right)},\;\;\;q=1,\ldots ,Q,\;\left|\delta_{n}^{\left(G\right)}\right|\leq \beta ,\;\;\;\;\;\;\;\;n=1,\ldots ,N,$$

where $w^{\left(q,G\right)}$ denotes the q-th column of $W^{\left(G\right)}$. Problem (13) remains a second-order-cone program. As in Section 3.1, aperture-range constraint (10) and minimum-spacing constraint (11) act only on the shared positions and can therefore be appended to (13) without modification when the actual application requires them. The position update, the convergence criterion, and the row-wise element-removal rule (a row is removed when $∥w_{row,n}{∥}_{2}<\nu$) are identical to those of Section 3.1.

### 3.3. Planar Sparse Arrays

The framework developed so far carries over from one dimension to two with only minor modification, the essential difference being that each element now moves in a plane rather than along a line. For a planar array, the perturbation of the n-th element is the vector $\delta_{n}={\left(\delta_{x,n},\delta_{y,n}\right)}^{T}$ acting on the position $r_{n}={\left(x_{n},y_{n}\right)}^{T}$, and the steering phase is given by (3). The first-order expansion (5) now acts on both coordinates, and the linearized array factor of the planar array becomes

(14) $$AF\left(\theta \right)\;\approx \;a^{\left(G\right)}{\left(\theta \right)}^{H}w^{\left(G\right)}+b_{x}^{\left(G\right)}{\left(\theta \right)}^{H}\left(w^{\left(G-1\right)}⊙\delta_{x}^{\left(G\right)}\right)+b_{y}^{\left(G\right)}{\left(\theta \right)}^{H}\left(w^{\left(G-1\right)}⊙\delta_{y}^{\left(G\right)}\right),$$

where $b_{x}\left(\theta \right)$ and $b_{y}\left(\theta \right)$ are, respectively, the first and second row vectors of the matrix $b\left(\theta \right)={\left[\;jke^{jk\cdot r_{1}},\ldots ,jke^{jk\cdot r_{N}}\right]}^{T}$, i.e., the components of $b\left(\theta \right)$ along the x- and y-axes, and $\delta_{x}$ and $\delta_{y}$ are the first and second row vectors of δ, i.e., the components of the perturbation along the x- and y-axes. The convex model solved at iteration G for the planar sparse array is therefore

(15) $$\underset{\delta^{\left(G\right)},\;w^{\left(G\right)}}{min}\;\;{∥\alpha^{\left(G\right)}⊙w^{\left(G\right)}∥}_{1}\;s.t.\;\;\;\left|a^{\left(G\right)}{\left(\theta_{i}\right)}^{H}w^{\left(G\right)}+b_{x}^{\left(G\right)}{\left(\theta_{i}\right)}^{H}\left(w^{\left(G-1\right)}⊙\delta_{x}^{\left(G\right)}\right)+b_{y}^{\left(G\right)}{\left(\theta_{i}\right)}^{H}\left(w^{\left(G-1\right)}⊙\delta_{y}^{\left(G\right)}\right)-F_{d}\left(\theta_{i}\right)\right|\leq \varepsilon ,\;\;\;\theta_{i}\in \Theta_{ML},\;\left|a^{\left(G\right)}{\left(\theta_{j}\right)}^{H}w^{\left(G\right)}+b_{x}^{\left(G\right)}{\left(\theta_{j}\right)}^{H}\left(w^{\left(G-1\right)}⊙\delta_{x}^{\left(G\right)}\right)+b_{y}^{\left(G\right)}{\left(\theta_{j}\right)}^{H}\left(w^{\left(G-1\right)}⊙\delta_{y}^{\left(G\right)}\right)\right|\leq \rho ,\;\;\;\theta_{j}\in \Theta_{SL},\;{∥\delta_{n}^{\left(G\right)}∥}_{\infty }\leq \beta ,\;\;\;\;\;\;\;\;n=1,\ldots ,N,$$

where the angle sets are sampled over the two-dimensional visible region $\left(\theta ,\phi \right)$, and the reweighting update (8), the position update, the convergence test on $\gamma =∥r^{\left(G+1\right)}-r^{\left(G\right)}{∥}_{1}$, and the element-removal threshold ν are unchanged with respect to Section 3.1.

As in the linear case, geometric constraints are appended to (15) only when the actual application requires them. The aperture-range constraint keeps the form $∥r_{n}^{\left(G\right)}+\delta_{n}^{\left(G\right)}{∥}_{\infty }\leq D/2$, where n=1,…,N, and is linear in the perturbations. The minimum-spacing requirement, however, deserves special attention for the more complicated situation of planar arrays: the elements are not ordered, so the spacing must be enforced between every pair of distinct elements n≠m as $∥\left(r_{n}+\delta_{n}\right)-\left(r_{m}+\delta_{m}\right){∥}_{2}\geq d$, which is non-convex because it lower-bounds a norm. Squaring the distance, expanding it around the current positions by a first-order Taylor expansion, and dropping the second-order term $∥\delta_{n}-\delta_{m}{∥}_{2}^{2}$—which is negligible under the trust-region bound (6)—converts this non-convex squared-distance constraint into the linear, and hence convex, constraint

(16) $${∥r_{n}^{\left(G\right)}-r_{m}^{\left(G\right)}∥}_{2}^{2}+2{\left(r_{n}^{\left(G\right)}-r_{m}^{\left(G\right)}\right)}^{T}\left(\delta_{n}^{\left(G\right)}-\delta_{m}^{\left(G\right)}\right)\;\geq \;d^{2},\;\;\forall \;n\neq m,$$

where n and m are the indices of any two different antenna elements of the array, D is the upper bound of the array aperture, and d is the lower bound of the minimum element spacing. When required by the radiator size or by mutual-coupling considerations, constraint (16) is appended to (15) while preserving convexity. The planar model can also be combined with the group-sparse objective of Section 3.2 to synthesize multiple-pattern planar arrays.

### 3.4. Incorporating a Minimum-Directivity Constraint

In gain-critical systems such as phased-array radar and satellite links, reducing the number of elements must not come at the price of an unacceptable loss of directivity, which is otherwise seldom controlled explicitly in sparse synthesis [25]. The proposed framework can additionally enforce a lower bound on the directivity without leaving the convex setting. For an array steered to the direction $\left(\theta_{0},\phi_{0}\right)$, the directivity can be expressed as the ratio of two Hermitian quadratic forms of the excitation vector [26,27],

(17) $$D\left(\theta_{0},\phi_{0}\right)\;=\;\frac{w^{H}A\;w}{w^{H}B\;w},$$

where the Hermitian matrices A and B are determined by the steering direction and by the integral of the array factor over the visible region, and their entries depend only on the inter-element distances; for isotropic elements the $\left(m,n\right)$ entry of the directivity matrix B is $B_{mn}=sinc\left(2\;∥r_{m}-r_{n}{∥}_{2}/\lambda \right)$, with $sinc\left(x\right)=sin\left(\pi x\right)/\left(\pi x\right)$. At iteration G, the matrix $B^{\left(G\right)}$ is evaluated at the current element positions $r^{\left(G\right)}$ and held fixed during the convex solve in the same spirit as the steering matrices $a^{\left(G\right)}$ and $b^{\left(G\right)}$, and a small eigenvalue flooring is applied to keep it positive semidefinite. Normalizing the mainbeam response to unity at the steering direction makes the numerator of (17) equal to one, so a minimum-directivity requirement $D\left(\theta_{0},\phi_{0}\right)\geq \xi$ reduces to the convex quadratic constraint

(18) $${\left(w^{\left(G\right)}\right)}^{H}B^{\left(G\right)}\;w^{\left(G\right)}\;\leq \;1/\xi ,$$

where ξ is the prescribed minimum directivity at the linear scale. Because $B^{\left(G\right)}$ is positive semidefinite, constraint (18) is convex and can be appended to any of models (9), (13), or (15); combined with convexified minimum-spacing constraint (16), it allows the joint control of sparsity, sidelobe level, aperture, element spacing, and directivity within a single quadratically constrained second-order-cone program. As shown in Section 4.4, this makes it possible to enforce a guaranteed minimum element spacing together with a directivity bound—a combination that the discrete-search directivity-constrained synthesis of [25] could not impose.

## 4. Numerical Results

The four examples are arranged as a progression of increasing structural complexity, each adding exactly one ingredient of the unified framework in Section 3. Example 4.1 only exercises the basic continuous position–excitation model (Section 3.1); Example 4.2 adds the reweighted ℓ_2,1_ group-sparsity constraint for a shared multiple-pattern layout (Section 3.2); Example 4.3 extends the model to planar geometry with the convexified minimum-spacing constraint (Section 3.3); and Example 4.4 further imposes the minimum-directivity constraint (Section 3.4) to exercise the full quadratically constrained model.

All the convex programs were modeled with CVX [29] and solved on a standard desktop computer. Unless otherwise stated, the stabilization factor was set to $\eta =10^{-3}$, the perturbation bound to β = 0.1λ, and the element-removal threshold to ν = 10^−3^ relative to the peak excitation magnitude.

### 4.1. Sparse Linear Array

The first example considers the synthesis of a linear array radiating a broadside flat-top shaped beam over an available aperture of 8λ. The desired pattern is unity in the flat-top region |u| ≤ 0.30 (with u = sinθ) and it is allowed a ripple of ±1 dB; the sidelobe region |u| ≥ 0.45 is constrained below a peak sidelobe level of −35 dB; and the transition band 0.30<|u|<0.45 is left unconstrained. The visible region is sampled uniformly with a step Δu = 0.005. The proposed method is initialized with N = 17 elements placed on a 0.5λ uniform grid and only aperture-range constraint (10) with D = 8λ is appended so that the inter-element spacing is left free; the reweighting stabilization factor is η = 0.015. For reference, the fixed-grid reweighted ℓ_1_ method of [13] is run on the same mask with a dense candidate grid of 0.01λ spanning the same 8λ aperture.

**Figure 2:**
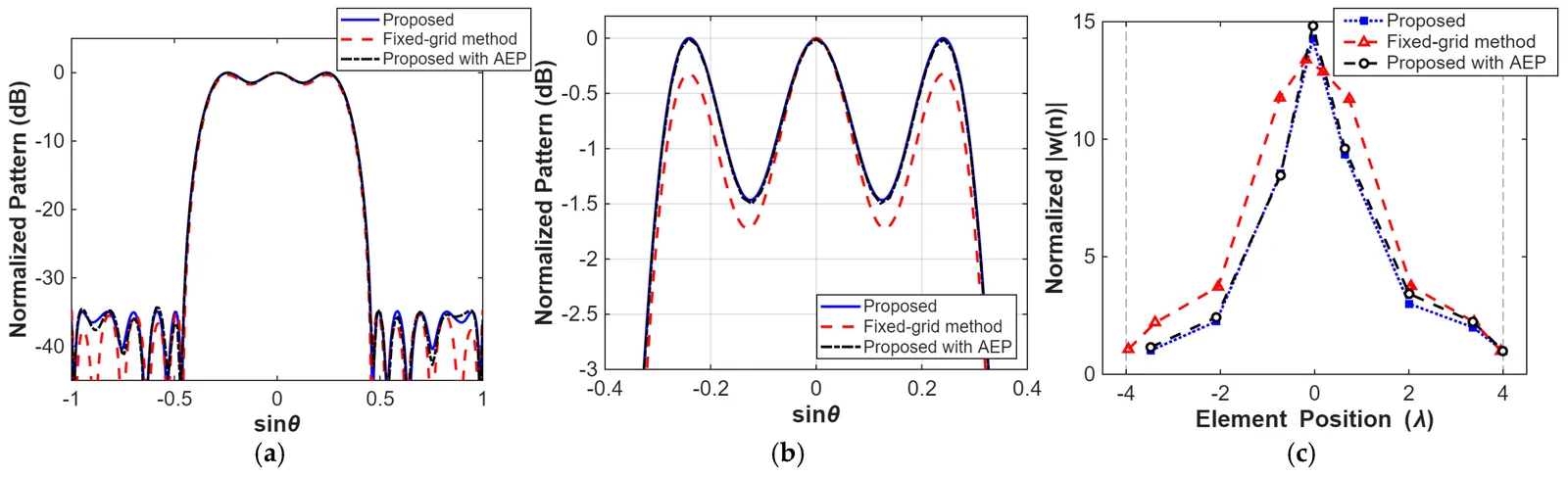
Synthesis results and full-wave simulation verification for the sparse linear array: (**a**) full radiation patterns of the proposed method, the fixed-grid method [13], and the HFSS full-wave simulation considering AEP; (**b**) zoomed-in views of the flat-top mainlobe comparing the proposed method, the fixed-grid method [13], and the HFSS simulation considering AEP; (**c**) final element positions and normalized excitation amplitudes.

Figure 2 shows the optimized radiation pattern and the final element layout, and compares the convergence of the element number with the fixed-grid reweighted l_1 method of [13]. As summarized in Table 1, the proposed method satisfies the flat-top mask with only eight elements, whereas the fixed-grid reweighted ℓ1 method of [13] needs ten elements for the same specification, a 20% reduction in the element count. Because the proposed method optimizes the positions in the continuum rather than selecting them from a fixed grid, it also yields a more compact 7.48λ aperture and a larger minimum element spacing of 0.64λ against 7.92λ and 0.36λ for [13]; the sub-wavelength clustering produced by the dense grid is therefore avoided even though no explicit spacing constraint was imposed. Starting from the 0.5λ uniform layout, the effective element number stabilizes within a few reweighting iterations, and the whole synthesis takes 1.94 s versus 15.93 s for the grid-based design, with the speed-up reflecting the much smaller number of optimization variables (one position and one excitation per physical element instead of one excitation per dense grid point). Furthermore, based on the optimized element positions, with the inclusion of the HFSS full-wave simulation considering the active element pattern (AEP), the results demonstrate favorable performance under the influence of mutual coupling and practical electromagnetic effects.

### 4.2. Multiple-Pattern Linear Array

The second example synthesizes a shared sparse layout that radiates Q=3 patterns: a pencil beam, a flat-top shaped beam and a cosecant beam. The three patterns share a common physical layout and differ only in their excitation columns. They are specified at the carrier frequency f_0_ = 10 GHz over an initial aperture of 9.5λ that is densely seeded with N = 134 candidate elements at λ/14 spacing, and the pattern is sampled in θ from −90° to 90° with a 0.2° step. The flat-top pattern requires unity response in |θ| ≤ 15° with sidelobes for |θ| > 25°; the cosecant-squared pattern follows a csc^2^-shaped mainlobe over 1° ≤ θ ≤ 30° with sidelobes for θ < −10° or θ > 40°; and the focused pattern is steered to broadside with sidelobes for |θ| > 10°. A common peak sidelobe level of −20 dB and a mainlobe matching tolerance of ε = 0.01 are imposed on all three patterns. The element count of the shared layout is measured by reweighted ℓ_2,1_ group-sparsity objectives (12) and (13), so that an element is removed only when its excitations vanish simultaneously in all three modes. The proposed joint method, which continuously adjusts the shared positions, is compared with the fixed-grid method of [23] solved on the same λ/14 grid.

**Figure 3:**
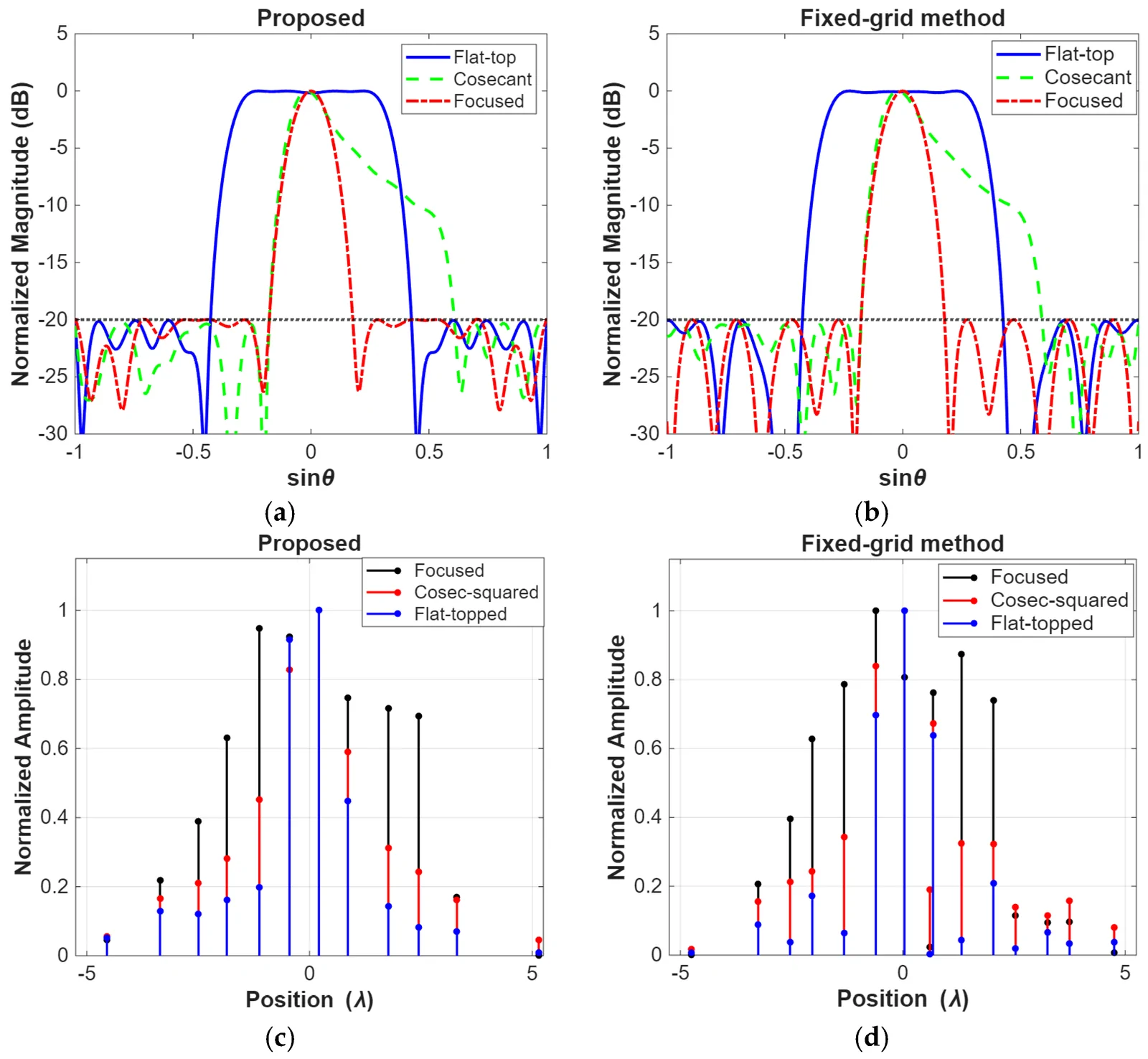
Synthesis results for the multiple-pattern linear array: (**a**) the three patterns synthesized by the proposed method versus their masks; (**b**) the three patterns synthesized by the fixed-grid method [23] versus their masks; (**c**) the shared element layout and normalized excitation amplitudes of the three operating modes for the proposed method; (**d**) the shared element layout and normalized excitation amplitudes of the three operating modes for the fixed-grid method [23].

As shown in Figure 3, all three masks are met simultaneously by a single shared layout: the flat-top and cosecant-squared patterns hold their prescribed mainlobe shapes and the focused pattern keeps its broadside peak, while every pattern respects the common −20 dB sidelobe bound. Starting from the 134-element dense points, the reweighted group-sparsity objective drives whole rows of the excitation matrix to zero, so that the same physical elements are switched off in all three modes. Because the proposed method relocates the surviving elements continuously rather than restricting them to the λ/14 grid, it converges to a sparser shared layout of 12 elements against 15 elements for the fixed-grid method under identical specifications, confirming that the continuous-position advantage observed in the single-pattern case (Section 4.1) carries over to the pattern-reconfigurable setting.

### 4.3. Planar Sparse Array

The third example considers a planar array over a square aperture of 5λ × 5λ radiating a broadside pencil beam, with convexified minimum-spacing constraint (16) appended to account for the physical size of the radiators. The mainbeam is normalized to unity at boresight (u = v = 0) and the sidelobe region r = √(u^2^ + v^2^) ≥ 0.25 is constrained below a peak sidelobe level of −17.3 dB, with the (u,v) plane being sampled over the visible region with a 0.05 step. The proposed method starts from an 11 × 11 = 121-element uniform grid at 0.5λ spacing and enforces a global minimum spacing of d = 0.25λ between every pair of elements through (16), whereas the fixed-grid reweighted ℓ_1_ method of [13] selects elements from a denser 21 × 21 = 441-point grid at 0.25λ spacing with no spacing control.

**Figure 4:**
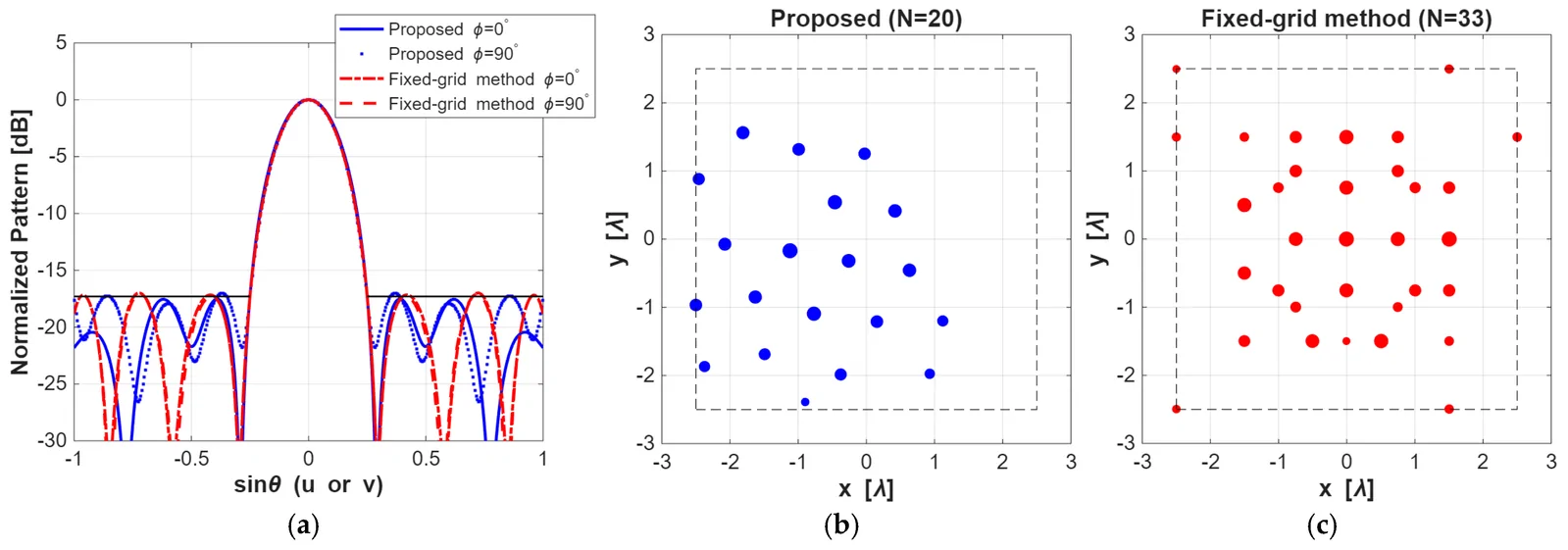
Synthesis results for the planar sparse array: (**a**) the proposed method and the fixed grid [13] method cut the principal plane direction diagram at ϕ = 0°and ϕ = 90°; (**b**) final element layout of the proposed method; (**c**) final element layout of the fixed-grid method [13].

The synthesized patterns and the resulting element layouts of both methods are shown in Figure 4, and the corresponding element counts and spacings are summarized in Table 2. As reported in Table 2, the proposed method meets the sidelobe mask with 20 elements over the 5λ × 5λ aperture and a minimum spacing of 0.65λ, whereas [13] requires 33 elements and produces densely clustered pairs separated by only 0.35λ; the two designs have comparable solution times (32.01 s versus 32.22 s), but the continuous-position model removes roughly 40% of the elements while guaranteeing a physically realizable spacing.

### 4.4. Directivity-Constrained Planar Array

The fourth example demonstrates the directivity-constrained variant of Section 3.4 and highlights the additional capabilities of the proposed framework by reproducing the planar benchmark of [25] and adding a practical element spacing constraint that the reference method did not consider. Following [25], a pencil beam with a peak sidelobe level below −25 dB is synthesized over a square aperture of 8λ×8λ, starting from a 17×17=289-element uniform grid with 0.5λ spacing and a minimum directivity of 23.52 dBi; the sidelobe region is $\left\{\left(u,v\right)\;|\;0.164^{2}<u^{2}+v^{2}\leq {\left[1+sin57{}^{\circ}\right]}^{2}\right\}$. The sidelobe mask and directivity bound are imposed through (15) and (18), respectively, and, in contrast to [25], while the minimum element spacing is initially relaxed, a global minimum element spacing of 0.43λ is ultimately enforced between every pair of elements through convexified constraint (16).

**Figure 5:**
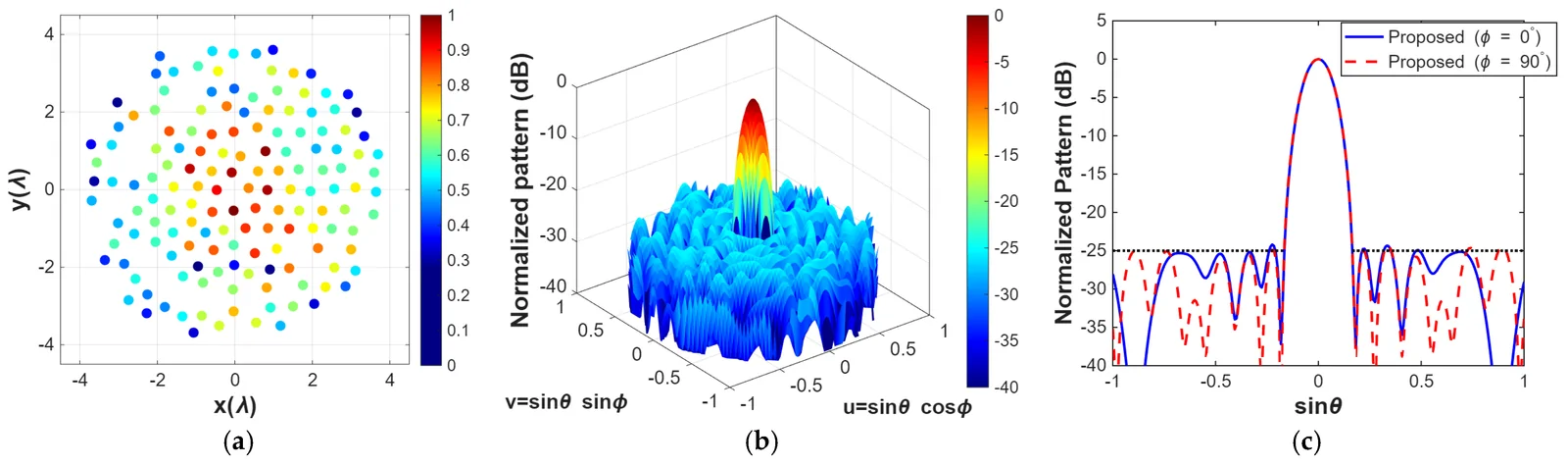
Synthesis results for the directivity-constrained planar array: (**a**) final element layout with excitation amplitudes; (**b**) 3D normalized radiation pattern; (**c**) 2D pattern cuts at *ϕ* = 0° and *ϕ* = 90° versus the mask.

The optimized array layout as well as the corresponding radiation pattern are presented in Figure 5. Table 3 compares the proposed method with the steerable nonsuperdirective convex synthesis of [20], the iterative convex method of [25], and the discrete inflating–deflating exploration algorithm (IDEA) of [28]; the values for [20,25,28] are taken from the reported results. The proposed method satisfies the same −25 dB sidelobe mask, the 57° scan requirement, and the 23.52 dBi minimum-directivity bound with 148 elements, one fewer than the 149 elements of IDEA and markedly fewer than the 163 elements of the steerable design of [20]. The synthesized layout attains a boresight directivity of 23.52 dBi, equal to the best reported value, while, unlike all three reference methods, guaranteeing a minimum element spacing of at least 0.43λ by construction through (16). At the 57° scan limit, the directivity remains above 20.9 dBi, while satisfying the 0.43λ minimum spacing requirement. Furthermore, when the minimum element spacing constraint is relaxed to 0.35λ, the proposed method yields an even smaller element count of 146.

## 5. Conclusions

A unified iterative convex-optimization framework has been presented for the synthesis of highly sparse arrays with joint continuous optimization of the element positions and the complex excitation amplitudes and phases. The framework combines a first-order Taylor expansion of the position perturbation under a trust-region constraint with an iteratively reweighted $l_{1}$ relaxation of the element-count objective, and each iteration reduces to a second-order-cone program; aperture and minimum-spacing constraints are formulated as convex constraints that are appended to the model only when the actual application requires them. The same machinery extends naturally to multiple-pattern linear arrays through a reweighted $l_{2,1}$ group-sparsity objective with a shared layout, and to planar arrays through a convexified pairwise minimum-distance constraint. A minimum-directivity requirement is then incorporated as a convex quadratic constraint, so that sparsity, sidelobe level, aperture, element spacing, and directivity are all controlled within a single model. Because the element positions are optimized in the continuum, the resolution of the method is no longer limited by a candidate grid, while the trust-region bound β offers a transparent trade-off between convergence speed and the accuracy of the linearization. By controlling sparsity, sidelobe level, aperture, element spacing, and directivity within a unified iterative convex framework, the proposed method provides a flexible and efficient tool for the design of compact, low-complexity, sparse antenna arrays, especially for integrated microwave and millimeter-wave front-end systems with constrained aperture and feeding resources.

## Figures and Tables

**Table 1:** Comparison of the proposed method with previously published methods for the linear flat-top shaped-beam example.

Method	Number of Elements	Aperture (λ)	Minimum Spacing (λ)	Times (s)
Fixed-grid reweighted ℓ1 [13]	10	7.92	0.36	15.93
Proposed method	8	7.48	0.64	1.94

**Table 2:** Comparison of the proposed method with previously published methods for the planar example.

Method	Number of Elements	Aperture (λ)	Minimum Spacing (λ)	Times (s)
Fixed-grid reweighted ℓ1 [13]	33	5 × 5	0.35	32.22
Proposed method	20	5 × 5	0.65	32.01

**Table 3:** Comparison of the proposed method with the steerable nonsuperdirective synthesis of [20], the directivity-constrained synthesis of [25], and the IDEA method [28] for the 8λ×8λ planar benchmark (SLL ≤−25 dB, ξ=23.52 dBi).

Method	No. of Elements	D(0°,0°) (dBi)	Min. Spacing (λ)
Convex method [20]	163	23.10	—
Iterative convex method [25]	148	23.52	0.43
IDEA [28]	149	23.40	—
Proposed method	148	23.52	≥0.43 (enforced)
	146	23.52	≥0.35 (enforced)

## Data Availability

The data presented in this study are available on request from the corresponding author due to restrictions related to the collaborative nature of the project.
